# Uncertainty of methane emissions coming from the physical volume of plant biomass inside the closed chamber was negligible during cropping period

**DOI:** 10.1371/journal.pone.0256796

**Published:** 2021-09-20

**Authors:** Ji Yeon Lim, Song Rae Cho, Gil Won Kim, Pil Joo Kim, Seung Tak Jeong

**Affiliations:** 1 Division of Applied Life Science (BK 21+ Program), Gyeongsang National University, Jinju, Republic of Korea; 2 Soil & Fertilizer Management Division, National Institute of Agricultural Science, Wanju, Republic of Korea; 3 Institute of Agriculture and Life Sciences, Gyeongsang National University, Jinju, Republic of Korea; 4 Horticultural and Herbal Crop Environment Division, National Institute of Horticultural and Herbal Science, Rural Development Administration (RDA), Wanju, Korea; Universidade Federal de Santa Maria, BRAZIL

## Abstract

In rice paddy, the closed chamber method is broadly used to estimate methane (CH_4_) emission rate. Since rice plants can significantly affect CH_4_ production, oxidation and emission, rice plantation inside the chamber is standardized in IPCC guidelines. Methane emission rate is calculated using the increased concentration inside the headspace. Biomass growth might decrease the headspace volume, and thus CH_4_ emission rates might be overestimated. To evaluate the influence of chamber headspace decreased by rice plant development on CH_4_ emission rates, five Korean rice cultivars were cultivated in a typical rice paddy, and physical volume changes in rice biomass were assayed using water displacement method. The recommended acrylic closed chambers (H. 1.2 m x W. 0.6 m x L. 0.6 m) were installed, and eight rice plants were transplanted inside the chamber with the same space interval with the outside. Biomass growth significantly decreased the headspace volume of the chamber. However, this volume covered only 0.48–0.55% of the closed chamber volume at the maximum growth stage. During the whole cropping period, mean 0.24–0.28% of chamber headspace was allocated by plant biomass, and thus this level of total CH_4_ emissions was overestimated. However, this overestimation was much smaller than the errors coming from other investigation processes (i.e., chamber closing hour, temperature recording, inconstant flooding level, different soil environments, etc.) and rice physiological changes. In conclusion, the influence of physical biomass volume inside the closed chamber was negligible to make the error in total CH_4_ emission assessment in rice paddies.

## Introduction

Methane (CH_4_) is the second potent greenhouse gas (GHG) after carbon dioxide (CO_2_) and contributed to approximately 18% of total global warming over the last 50 years [1]. Over 60% of the total CH_4_ emissions originated from human activities like agriculture, industry, and waste management [2]. In particular, the agriculture sector solely contributes to almost half of the anthropogenic CH_4_ emissions, including rice production which accounts for more than 10% of anthropogenic sources [3].

Methane exists in the rice fields either as gas or in the dissolved phase [4,5]. However, dissolved CH_4_ concentration is minimal due to its low water solubility and the lack of ionic form [6]. Three possible mechanisms are known as the CH_4_ emission pathway from rice paddy soil to the atmosphere namely, diffusion, ebullition, and plan-mediated transport. However, rice plant-mediated transport via aerenchyma tissue is accepted as the main CH_4_ emission route in rice fields [4,7,8]. This pathway is known to cover approximately 80–90% of CH_4_ emission from rice cropping fields [9–12].

To estimate CH_4_ emission rates in rice cropping fields, two different methods such as closed-chamber and micrometeorological techniques (e.g., eddy covariance or gradient techniques) were generally used [13–15], but the former is mainly utilized since it is relatively easier and less expensive for installation and operation. Transparent materials like acrylic sheet, perspex or rigid plastics are commonly used to make the closed chamber having the dimension big enough to cover rice plants [16–18]. In addition, since CH_4_ is transported from the rhizosphere to the atmosphere via rice aerenchyma tissues [19], rice planting inside the chamber is recommended to have equal space with the outside [18,20].

Gas samples are collected by closing the chamber lid for a period of time, and CH_4_ emission rate (mg m^-2^ h^-1^) is calculated by assaying the increased CH_4_ concentration inside the chamber [21]. The reliability of closed-chamber method in estimating CH_4_ emission rates has been verified through a number of studies [22–25]. Various designs of the chamber have been developed to stabilize microclimate conditions (e.g., light, wind, CO_2_ concentration, temperature, etc.) inside the chamber comparable to the outside condition [26–29].

Methane emission rate (F, mg m^-2^ h^-1^) is calculated using the increased CH_4_ concentration per hour inside the chamber [21]. In this equation, the headspace volume of the chamber was fixed without considering rice plant volume allocation. As rice plants matured, the developed rice biomass visually occupied a big part of chamber headspace, and then the void headspace appeared to become smaller. However, there was no study that evaluated the influence of physical allocation of rice plant biomass inside the closed chamber on the estimation of CH_4_ emission rates during rice cultivation.

We hypothesized that as rice plants grow, developed plant biomasses occupy significant headspace volumes of chambers, and thus total CH_4_ emissions (kg ha^-1^) might be overestimated due to use of fixed chamber volume in calculation rather than the real headspace volume. In this field test, to evaluate the influence of rice biomass allocation inside the closed chamber on uncertainty of CH_4_ emission rates, the physical volumes of five different rice cultivars were periodically evaluated using water displacement method during rice cultivation period. The uncertainty of total CH_4_ emissions which come from the decrease of chamber volume with plant biomass developing was evaluated.

## Materials and methods

### Experimental site selection

The experimental plots were installed at the agronomic rice field of *Gyeongsang* National University (35°08′56′′N and 128°05′46′′E), Jinju, South Korea. The region has a typical temperate monsoon climate. For the last 30 years, the mean temperature and annual precipitation were 13°C and 1513 mm, respectively [30]. The soil was classified as *Pyeongtaeg* series (fine-silty, mixed, nonacid, mesic Typic haplaquent) and the field was exclusively utilized for rice cultivation for over 40 years. Before the experiment, soil had slightly acidic pH (5.9±0.2, 1:5 with H_2_O) and low fertility (21.5±2.3 g kg^-1^ of organic matter, 0.71±0.06 g kg^-1^ of total N, and 41±4.1 mg kg^-1^ of available P).

### Rice cultivar selection and cultivation

To investigate the influence of rice plant biomass volume inside the closed chamber on calculating CH_4_ emission rates, five Korean rice cultivars (*Chuchung*, *Dongjin*, *Ilmi*, *Junam*, and *Saenuri*) were selected, and the physical volume changes of rice plants were periodically monitored during cropping period. The selected cultivars belong to *Japonica rice* and the late-maturing species (130–140 days of cultivation period).

Total 15 experimental plots (5 cultivars × 3 replications) having 100 m^2^ area per plot (10 m × 10 m size) were arranged in a randomized block design in the experimental fields. Twenty-one-day old seedlings were manually transplanted with a spacing of 30 cm × 15 cm. Single rice seedling per hill was manually transplanted to accurately compare the changes in plant biomass volume and harvested after 130 days.

Rice were cultivated under the same condition. Nutrients were managed by chemical fertilization (N-P_2_O_5_-K_2_O = 90-45-57 kg ha^-1^) based on the Korean fertilization standard for rice [31]. Weeds and pathogens were properly controlled. The irrigation water level was automatically controlled with a depth of 5–7 cm throughout cropping season, and water was drained four weeks before harvesting.

### Evaluation of rice plant biomass volume and soil properties

To determine the changes in headspace volume inside the closed chamber, rice plants outside the chamber were sampled total 12 times during cropping season (one-week interval during vegetative growth period from transplanting to panicle initiation stage, and thereafter two-weeks interval to harvesting stage). Five hills of rice plants per plot were sampled at every sampling stage. Whole rice plant (above- and below-ground biomass) were uprooted and kept in an icebox to minimize changes in physical biomass volume. The plant biomass above the flooded water level was carefully separated by distinguishing green and white color differences that represent the upper and lower parts of the irrigated water table, respectively. The physical volume of plant biomass was determined by the water displacement method, which was modified from the methodology to measure soil particle density [32]. Rice growth characteristics (i.e., tiller number per hill, plant height, and above-ground biomass productivity) were simultaneously evaluated base on the Korean rice sampling standard [33].

During rice cultivation period, soil redox potential (Eh value) was consistently monitored by using Eh meter. The electrodes were permanently installed at 10 cm of soil depth. Soil temperature was also monitored with a thermometer at every sampling.

### Gas sample collection and analysis

A closed-chamber method was used to estimate CH_4_ emission rates [16–18,20]. Three pairs of transparent acrylic chambers (length 60 cm × width 60 cm × height 120 cm) were permanently installed in each plot. Eight rice plants were transplanted inside the chambers with the same space interval (30 cm × 15 cm) on the outside. Static chambers were kept open during the entire cropping season, except for gas sampling. Each chamber contained two electric fans and a thermometer inside to mix air and monitor temperature.

Before starting regular gas samplings, the research protocol was established to set sampling conditions such as sampling hour, interval, and times. In the preliminary measurement, 30 minutes was selected as chamber lid closing hour to collect gas samples, since the positive linear relationships between CH_4_ concentration and chamber closing hour (0, 10-, 20-, 30-, and 60-min. chamber closing) were observed. Gas sampling time was fixed at 10:30 am, because the mean daily emission rate was detected in this time via 12 times of gas sampling a day. Gas samples were collected once a week during rice cultivation. At 0 and 30 min after chamber closing, gases were sampled from the headspace using a 50 mL gas-tight syringe and immediately transferred to a 30 mL vacuum glass vial.

Methane concentrations were analyzed using gas chromatography (Shimadzu, GC-2010 PLUS, Japan) equipped with Porapak NQ column (Q 80–100 mesh) and a flame ionization detector (FID). Column, injector and detector temperatures were controlled at 35, 200 and 250°C, respectively. Helium and hydrogen gases were used as carrier and combustion gases, respectively. Methane emission rate (F, mg m^-2^ h^-1^) was calculated using the increased CH_4_ concentration inside the chamber for a specific time interval (Eq 1) [21].

$$F\;(mg\;m^{‐2}\;h^{‐1})=(\Delta C/\Delta t)\times (V/A)\times \rho \times (273/T)$$ (Eq 1)

where ΔC is the increased CH_4_ concentration (mg m^-3^), Δt is the chamber closing hour, V is the chamber volume (m^3^), A is the chamber surface area (m^2^), ρ is the gas density (0.714 mg cm^-3^) of CH_4_ at the standard state, and T (K) is the absolute temperature (273 + temperature in the chamber,°C).

To assess the influence of real chamber headspace which might be reduced by plant biomass development on CH_4_ emission rates, net CH_4_ emission rate (F_i_, mg m^-2^ h^-1^) was calculated by the newly developed equation (Eq 2) following as.

$$F_{i}\;({\mathrm{mg}\;m}^{‐2}\;h^{‐1})=(\Delta C/\Delta t)\times V_{\mathrm{met}}/A\times \rho \times (273/T)$$ (Eq 2)

where V_net_ means the real headspace volume of chamber which considered rice plant biomass volume from the growth chamber volume.

The total CH_4_ emissions for the entire cropping period were calculated using the following formula (Eq 3) [34].

$$Total\;{\mathrm{CH}}_{4}\;emission\;(\mathrm{kg}\;{\mathrm{ha}}^{‐1})=\sum_{i}{}^{n}(R_{i}\times D_{i})$$ (Eq 3)

where R_*i*_ is the daily CH_4_ emission rate (g m^-2^ d^-1^) in the *i*^th^ sampling interval. R_*i*_ is calculated by multiplying CH_4_ emission rate (F & F_i_, mg m^-2^ h^-1^) and 24 hours. D_*i*_ is sampling day interval between the *i*^th^ and (*i-1*) ^th^ day. n is the number of samplings.

### Statistical analysis

The statistical analysis was carried out using the SPSS package (IBM SPSS Statistics 23). All datasets were subjected to variance analysis. Mean differences of plant volumes, growth characteristics, and total CH_4_ emissions among rice cultivars were assessed through one-way ANOVA, Tukey HSD test. The differences of total CH_4_ emissions between calculation methods (with and without plant volume consideration) were evaluated by independent-sample t-test. In order to estimate relationship between CH_4_ emission rates and variables, correlation and linear regression analyses were performed by Pearson correlation analysis (SPSS and Sigma Plot software).

## Results

### Changes in soil temperature and redox potential (Eh value)

**Fig 1:**
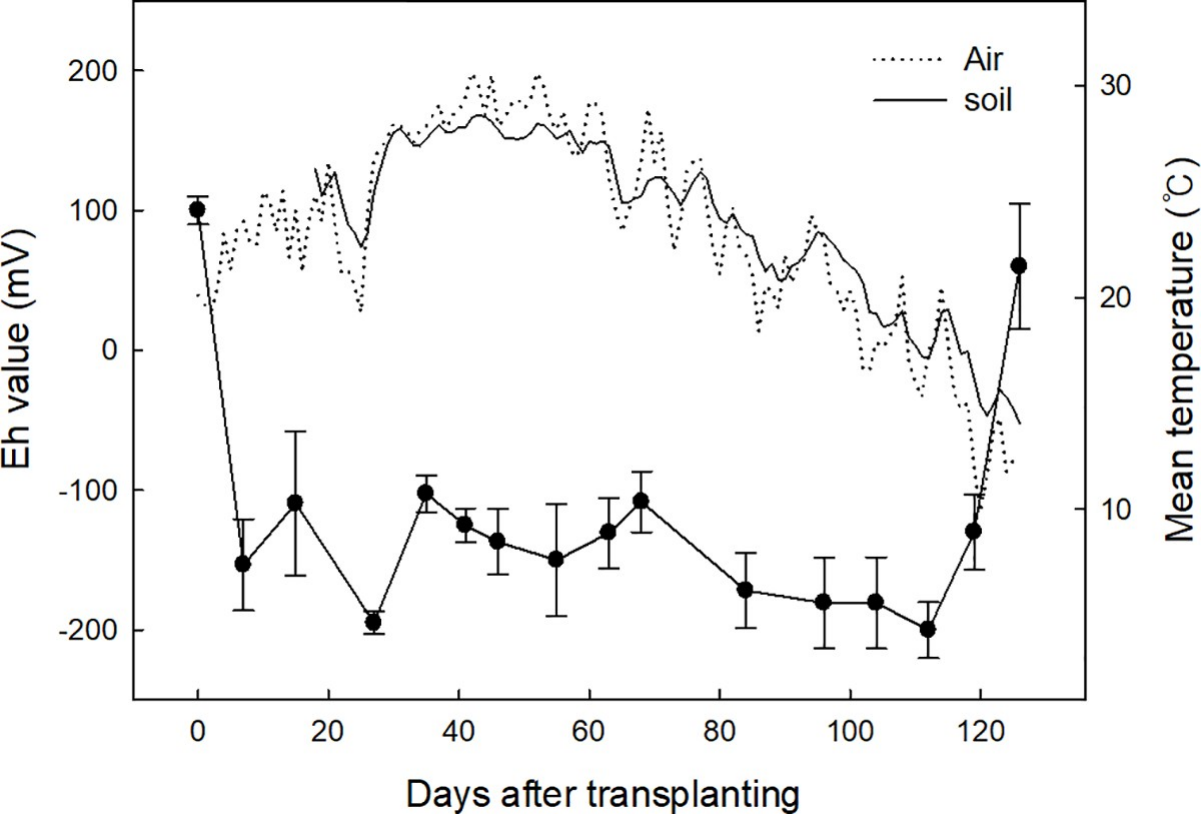
Changes in soil temperature and Eh values during rice cultivation. Vertical bars indicate standard deviation (n = 5).

Soil temperature was similarly changed with air temperature during rice cultivation (Fig 1). However, it was not different among the selected rice cultivars. Soil temperature increased after rice transplanting and peaked at approximately 25 to 60 days after transplanting, and thereafter, rapidly decreased.

Soil Eh values dramatically decreased to less than negative 150 mV within one week after rice transplanting (Fig 1). This anaerobic soil condition was maintained for over 100 days, but Eh values rapidly increased after drainage for harvesting. However, soil Eh values were not discriminated among rice cultivars.

### Changes in rice biomass volumes and growth characteristics

**Fig 2:**
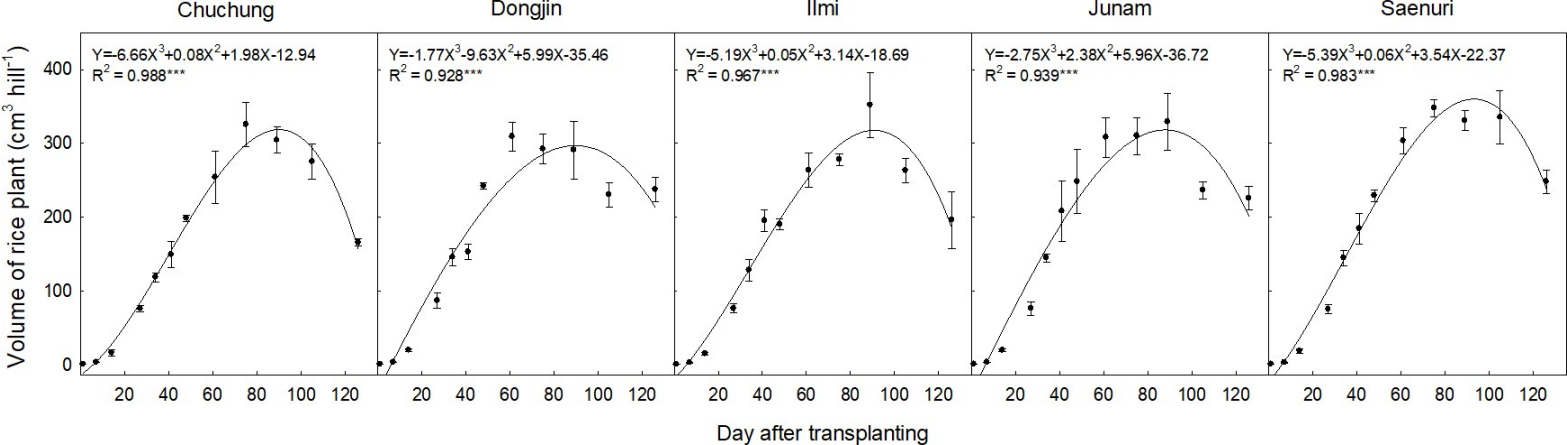
Changes in biomass volumes of selected rice cultivars during rice cropping season.

The biomass volume of rice cultivars was changed with a sigmoid pattern over the whole rice life cycle (Fig 2). After transplanting, the physical volume of rice plants gradually increased up to 90 days, and thereafter, it sharply decreased to the harvesting phase. The allocation of biomass volume inside the chamber was negligible at the early rice-growing stage but slightly increased with plant growth. It was maximized at the panicle initiation stage.

**Fig 3:**
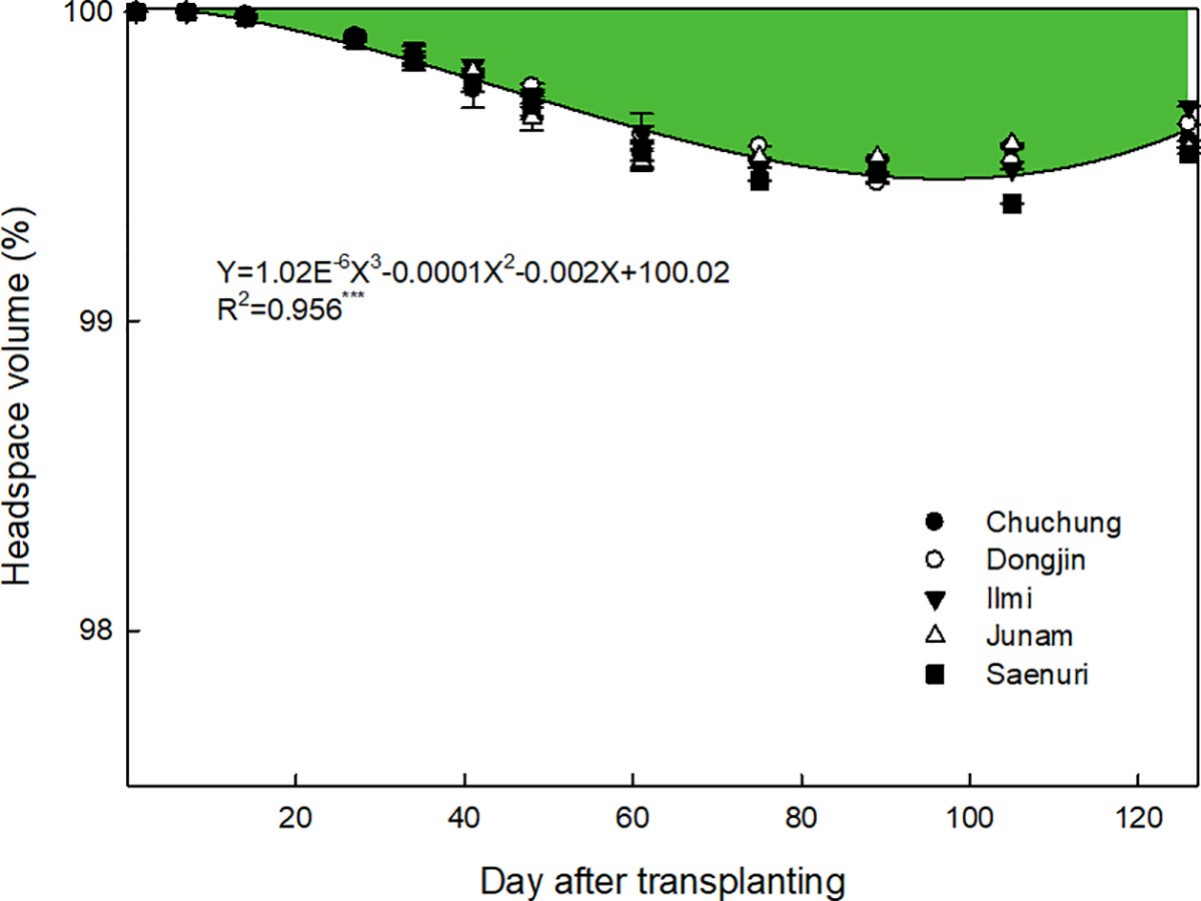
Changes in the headspace volume inside closed chamber during rice cultivation period. Green color indicates the allocation (%) of plant biomass inside the closed chamber headspace.

Single rice seedling was transplanted in this field test, but it propagated to the mean 8–11 tillers per hill at the early tillering stage (S2 Fig). In this period, the above-ground biomass of rice plants had 15–20 cm^3^ of physical volumes per hill, but only 40–45% (6–9 cm^3^ per hill) of biomass was placed over the flooded water table. Thus, the physical volume of eight rice plants inside the chamber was 48–72 cm^3^ and covered only 0.012–0.018% of the chamber headspace (Fig 3). Plant height and biomass productivity were gradually increased with plant growth (S2 Fig), and the above-ground biomass was maximized at the panicle initiation stage, having 309–352 cm^3^ of physical volume per hill. In this stage, approximately 80% (245–281 cm^3^ per hill) of above-ground biomass was placed above the flooded water table. Eight hills of rice plant inside the chamber had the maximum physical volume (1,960–2,248 cm^3^ over the water table) and occupied 0.48–0.55% of the chamber headspace. Thereafter, plant biomass volume slightly decreased with maturing to 166–248 cm^3^ per hill at the harvesting stage. A total of eight hills of rice plant inside the chamber had 1,328–1,984 cm^3^ of physical biomass volume and occupied approximately 0.31–0.46% of chamber headspace. As a result, the physical volume of rice plant biomass inside the chamber occupied average 0.24–0.28% of chamber headspace during the whole rice cropping season, and thus, this level of total CH_4_ emissions was overestimated.

**Fig 4:**
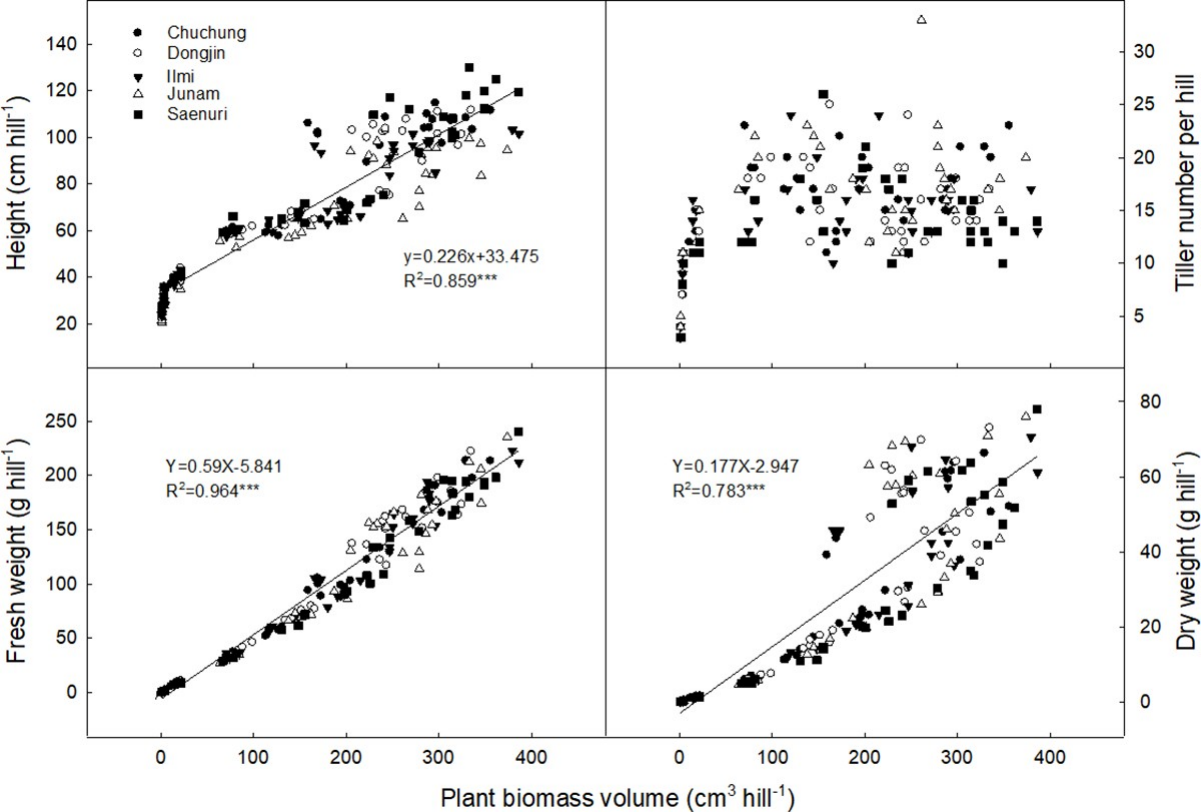
Relationships between rice plant biomass volume and rice growth characteristics during rice cropping period.

The physical rice growth characteristics (tiller number, plant height, fresh and dry weight) were gradually developed to the heading stage, and thereafter, slightly decreased towards the harvesting stage (S2 Fig). Single rice seedling was transplanted, but the number of tillers increased up to 20–22 per hill at the end of tillering stage. However, the effective tiller number decreased to 14–18 per hill at the harvesting stage. Plant height increased by the flowering stage and thereafter, stabilized. Fresh biomass weight was changed with sigmoid pattern, which was similar with plant volume changes. Among rice cultivars, these growth characteristics were not discriminated at early growing stage but showed significant difference at the maximum growth stage (S1 Table). However, plant volume and fresh weight were not significantly different among rice cultivars. The physical volume of rice plants showed highly positive correlation with plant height and biomass weight (fresh and dry) (Fig 4).

### Difference of methane emission rates with/without considering plant biomass volume inside the chamber

**Fig 5:**
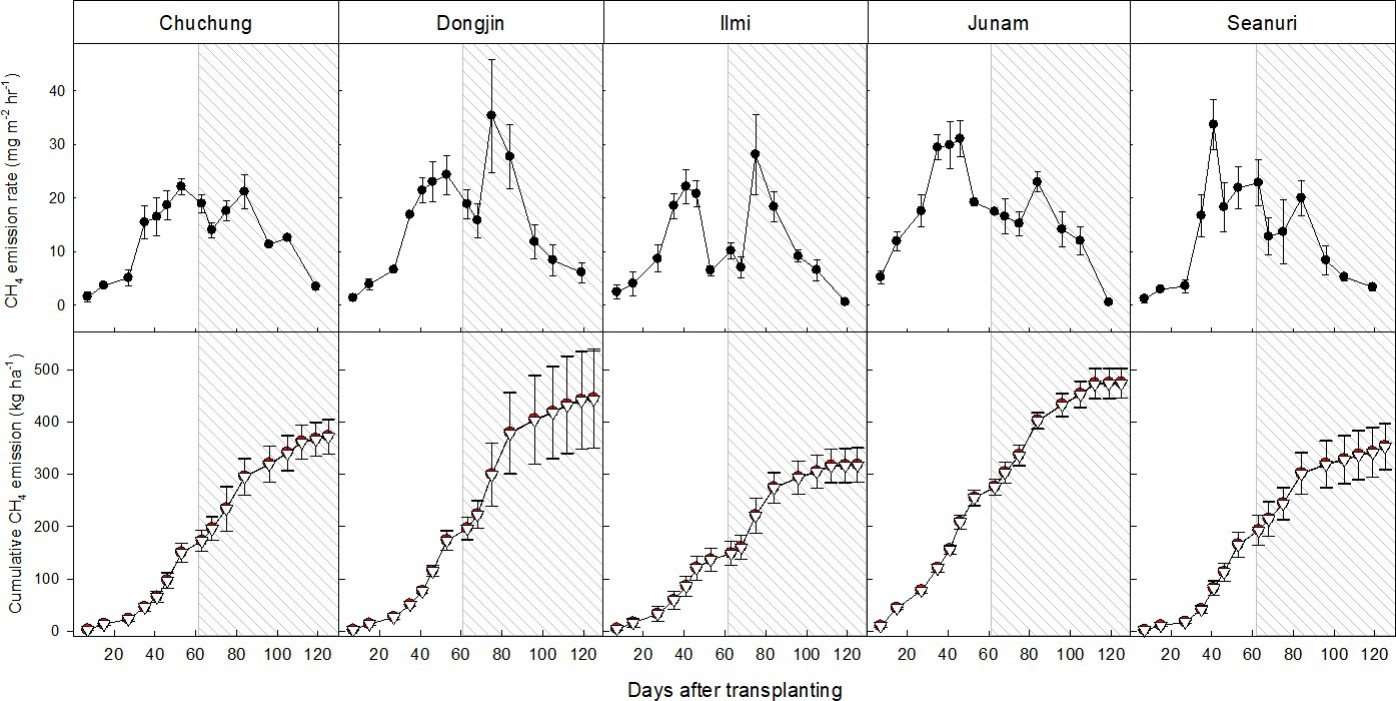
Changes in CH_4_ emission rates (above) and cumulative CH_4_ emissions (below) which were calculated by with (white triangle) and without (red circle) biomass volume consideration during rice cropping season. White and shaded areas indicate the vegetative and reproductive stage of rice plant, respectively.

Irrespective of rice cultivars, CH_4_ emission rates similarly changed during rice cultivation (Fig 5). Low CH_4_ emission rates were observed at the early growing stage, but it was highly increased with developing anaerobic soil condition and rice plants. Two peaks of CH_4_ emission rates were observed at approximately 50 and 80 days after transplanting in all rice cultivars. After panicle initiation stage, CH_4_ emission rates rapidly decreased with plant maturing to the background level at harvesting stage.

Total CH_4_ emissions were slightly different among the selected rice cultivars (Fig 5). *Junam* and *Dongjin* cultivar had the highest total CH_4_ emissions with 475 and 445 kg ha^-1^, respectively, and then followed by *Chuchung*, *Saenuri*, *and Ilmi cultivar* with 373, 354 and 318 kg ha^-1^ of total CH_4_ emissions, respectively. Among rice growth characteristics, the number of tillers showed a highly positive correlation with total CH_4_ emissions (Table 1).

**Table 1:** Correlation between total CH_4_ emissions and rice growth characteristics at harvesting stage.

Volume	Height	Tiller no.	Fresh biomass weight	Dry biomass weight
-0.518	-0.393	0.916***	-0.136	0.558

***Significant differences (P<0.001).

The uncertainty of total CH_4_ emissions coming from rice plant biomass occupying chamber headspace was negligible. The allocation of rice plant biomass clearly increased with plant growth (Fig 3), but this volume occupied only 0.48–0.55% of closed chamber headspace at the maximum plant biomass developing stage. This allocation decreased with maturing to 0.31–0.46% at the harvesting stage. During the whole rice cultivation period, rice plant biomass occupied only 0.24–0.28% of closed chamber headspace on average. Therefore, these small levels of total CH_4_ emissions might be overestimated more than the CH_4_ emissions conventionally calculated by the IPCC investigation protocol.

## Discussion

Methane is biologically formed from the anaerobic decomposition of organic matter [35,36]. Methane production is initiated under extremely reduced soil condition having negative 150–160 mV of soil redox potential (Eh value) [37,38]. Therefore, methanogenesis can be influenced mainly by organic substrates and oxygen contents in soil. In this field study, soil Eh values were dramatically decreased with flooding from 100 mV to negative 150 mV within a week after transplanting and stabilized within minus 180–220 mV until drainage for harvesting (Fig 1). However, we could not find any difference of soil Eh values between inside and outside the chamber.

In the flooded rice cropping fields, closed chamber techniques have broadly used to determine CH_4_ emission rates due to its minimal cost for installation and management, easy manipulation, and high efficiency to detect low CH_4_ fluxes [17,39–43]. To calculate CH_4_ emission rates in closed chamber method (Eq 1) [21], the increased CH_4_ concentration and temperature for chamber closing hour were considered as the variables. Chamber headspace volume was calculated by considering the flooded water level. In addition, gas density was designed as the fixed value. Rice plantation inside the chamber is essential to make the same condition with outside since rice plants strongly influence CH_4_ production and emission characteristics.

However, this closed chamber technique has several limitations in accurately characterizing CH_4_ emission rates, since the environment condition might be big different between inside and outside the chamber. Firstly, the net chamber headspace could be significantly decreased by plant biomass development, and therefore, the CH_4_ emission rates calculated by the fixed headspace volume considering only water table changes might be largely overestimated. However, we found the change of rice biomass volume negligibly influenced on evaluating CH_4_ emissions (Fig 5). During the whole rice cropping period, rice plant biomass occupied only 0.2–0.3% of closed chamber headspace, and therefore, only these levels of total CH_4_ emissions can be overestimated. This overestimation might be much less than the errors caused by other practices on chamber management (i.e., chamber closing hour, inconstant flooding level, temperature rising) and changes of plant mediating physiological characteristics.

**Fig 6:**
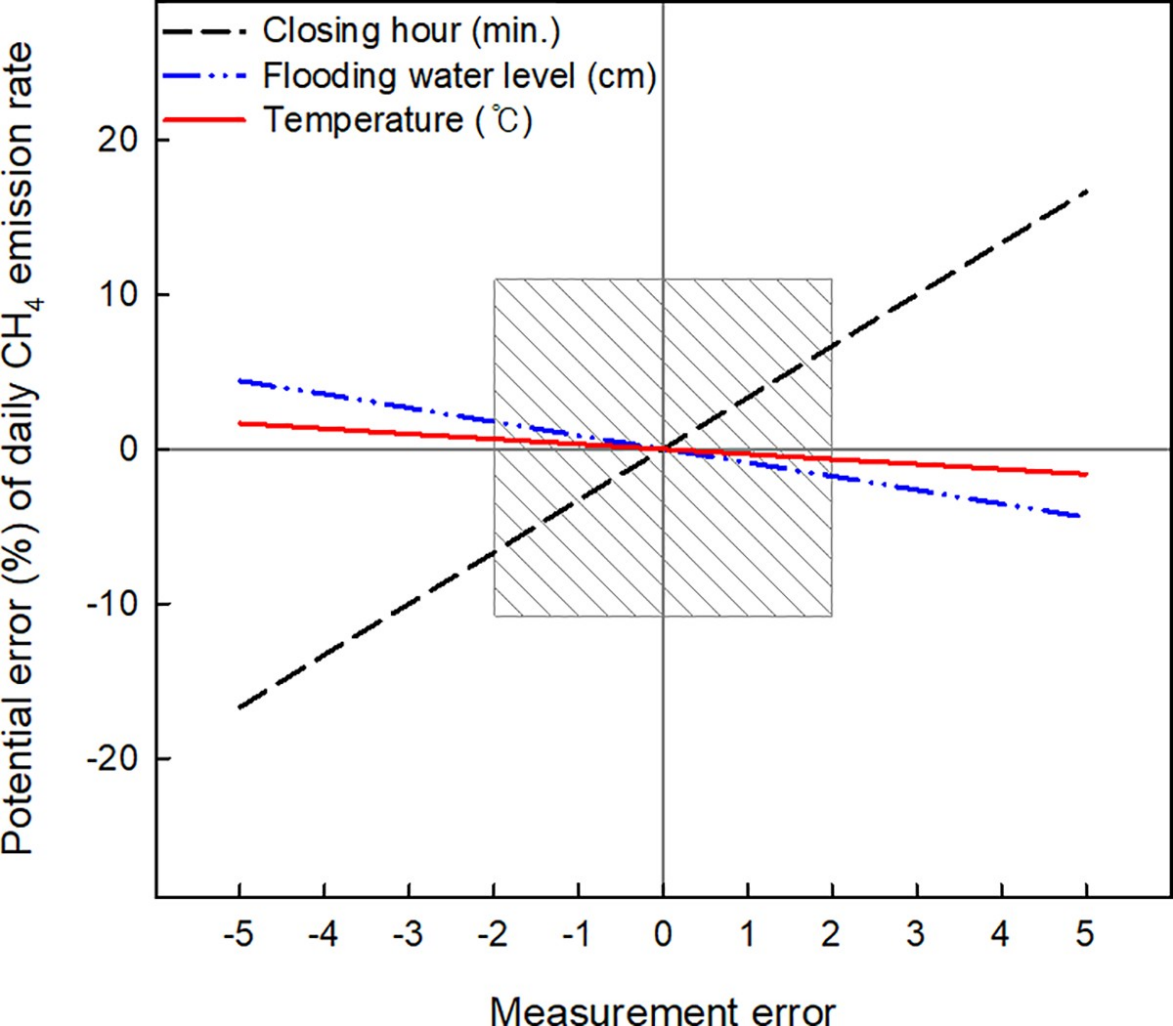
Potential error (%) in daily CH_4_ emission rates coming from chamber management practices during gas sampling. The shaded square box indicates the range of potential error produced by the measurement error of ± 2 minutes of closing hour, ± 2 cm of flooding water level, and ± 2°C of headspace temperature.

In the guidelines for measuring CH_4_ emission [18], chamber closing for 30 minutes is recommended, but the exact time control is difficult under the manual system with many chamber replications. For example, closing time error (longer or shorter closing) of 2 minutes can generate an error from approximately ±6.7% on a daily CH_4_ emission rate (Fig 6). In rice cropping fields, it is not easy to control the irrigated water level constantly even under automatically controlled irrigation systems. The flooded water table difference can change the headspace volume inside the chamber. Two cm over- or less measurement of flooding water table can make the error approximately ±1.77% on the daily CH_4_ emission rate. During this field investigation, the temperatures between inside and outside of the chamber showed a big difference on gas sampling stage, particularly in hot summer season (S1 Fig). Air temperature inside the closed chamber was average 5°C higher than the outside during the investigation period. The enhanced temperature was considered to calculate CH_4_ emission rates as a variable. However, 2°C over- or less measurement of headspace temperature at gas sampling time can generate approximately -0.65% to +0.66% of calculation error on the daily CH_4_ emission rate.

Furthermore, temperature increase inside the closed chamber could influence rice plant development and soil microbial characteristics which are fundamental traits to CH_4_ production and emission. In fact, at the harvesting stage, above-ground biomass productivity of rice in a closed chamber was minimum 5–10% higher than that of rice outside the chamber. Given that methanogenesis activity showed a very high positive correlation with soil temperature [44–47] and rice biomass productivity [48], the enhanced soil temperature and improved rice biomass productivity inside the chamber may significantly increase CH_4_ production and emission activity and then make a big difference in total CH_4_ emissions between inside and outside the chamber. However, the temperature effect induced by chamber has not yet been properly evaluated. It may be possible that the error from chamber enclosure is far more influential than chamber handling errors (i.e., closing time, water table, and temperature measurement).

The content and lability of soil organic matter might be similar between inside and outside the chamber, but rice root activity is probably much stronger inside than outside, which can be presumed from higher rice above-ground biomass development inside the chamber. In general, higher rice biomass can release more root exudates [49] and then produce more CH_4_ during rice cropping. Root exudates contain various metabolites such as organic acids (acetic, citric, oxalic acid, etc.), sugars (glucose, sucrose, etc.) and amino acids [50], and they can be important organic substrates for methanogens [51]. We confirmed that CH_4_ emission rates showed highly positive correlation with rice growth characteristics like biomass productivity (Table 2). The higher biomass inside the chamber might increase CH_4_ production by minimum 5–10% over out of the chamber. Furthermore, dead root biomass is also an important organic substrate for methanogenesis, so greater biomass yields inside the chamber can produce more organic residues thus increase CH_4_ production.

**Table 2:** Correlation coefficient between CH_4_ emission rates and rice growth characteristics at vegetative and reproductive stage.

Cultivar	Vegetative stage				Reproductive stage			
	Height	Till no.	Fresh weight	Dry weight	Height	Till no.	Fresh weight	Dry weight
Chuchung	0.848**	0.627	0.910***	0.914***	-0.889*	-0.012	-0.415	-0.396
Dongjin	0.824**	0.612	0.840**	0.837**	0.942**	0.353	0.839*	-0.443
Ilmi	0.605	0.817**	0.617	0.636	0.774	0.877*	0.448	-0.540
Junam	0.660	0.941***	0.600	0.552	0.875*	0.757	0.961**	-0.014
Saenuri	0.705*	0.714*	0.768*	0.781*	0.980***	-0.933**	-0.800	-0.970**
Sum	0.649***	0.728***	0.708***	0.696***	0.133	0.321	0.216	-0.366

Note

*, ** and *** denote significant differences at the 0.05, 0.01, and 0.001 probability levels, respectively.

Methane is emanated through three different pathways in rice fields: transport through aerenchyma channel, ebullition as gas bubbles, and diffusion through the flooded water and soil interfaces [52]. However, in the rice paddy, most of CH_4_ emitted is diffused via aerenchyma as both water-dissolved and gas phases from the flooded soils to the atmosphere [4,9,10,53]. Simply, we can think that rice plants with greater aerenchyma might emit more CH_4_ from rhizosphere soils to the atmosphere [9]. However, aerenchyma channel can also transport oxygen from the atmosphere to plant and rhizosphere [53,54]. Thus, at the rhizosphere of greater aerenchyma having rice, more CH_4_ may be oxidized by methanotrophs which use oxygen to metabolize CH_4_ as their carbon and energy source. However, the net effect of aerenchyma size on CH_4_ production and consumption was not clear yet. Therefore, we need to study more the influence of the chamber enclosure on methanogenesis to improve the accuracy of estimating CH_4_ emission rates in rice cropping fields.

## Conclusion

During the whole rice cropping period, rice plant biomasses occupied the mean 0.24–0.28% of chamber headspace volume, and thus this level of total CH_4_ emissions was overestimated in the conventional calculation (proposed by IPCC Guidelines). However, this overestimation was much less than the errors which can be generated from incorrect chamber managements such as closing time control, flooding water table management, temperature misreading, etc. Chamber effect which can increase temperature and differentiate plant and microbial activities inside the chamber might lead to more serious errors in estimating CH_4_ emissions in rice paddies. In conclusion, the uncertainty coming from the occupation of rice plant biomass volumes inside the closed chamber was negligible to estimate total CH_4_ emissions in rice paddy fields.

## Supporting information

S1 Fig Changes in air temperatures inside and outside the closed chamber during rice cropping period. (DOCX)

S2 Fig Changes in plant growth characteristics during rice cropping season. (DOCX)

S3 Fig Static chamber installation and rice plant growth inside the closed chamber. (DOCX)

S1 Table The maximum plant volumes and growth characteristics with statistical significance between rice cultivars. (DOCX)

S1 Data Changes in CH_4_ emission rates (mg m^-2^ hr^-1^) which were calculated without considering biomass volume inside chamber during rice cropping season. (XLSX)

S2 Data Changes in rice biomass volume (cm^3^ hill^-1^) during rice cultivation. (XLSX)

## References

[1] IPCC, 2014. Climate Change 2014: Synthesis Report. In: Pachauri R.K., Meyer L.A. (Eds.), Contribution of Working Groups I, II and III to the Fifth Assessment Report of the Intergovernmental Panel on Climate Change. IPCC, Geneva, Switzerland, p. 151. https://www.ipcc.ch/site/assets/uploads/2018/05/SYR_AR5_FINAL_full_wcover.pdf.

[2] Saunois M., Jackson R.B., Bousquet P., Poulter B., Canadell J.G., 2016. The growing role of methane in anthropogenic climate change. Environ Res Lett 11, 120207. 10.1088/17489326/11/12/120207.

[3] Yusuf R. O., Noor Z. Z., Abba A. H., Hassan M. A. A., Din M. F. M., 2012. Methane emission by sectors: a comprehensive review of emission sources and mitigation methods. Renew. Sustain. Energy Rev. 16(7), 5059–5070. 10.1016/j.rser.2012.04.008.

[4] Nouchi I., Mariko S., Aoki K., 1990. Mechanism of Methane Transport from the Rhizosphere to the Atmosphere through Rice Plants. Plant Physiol 94, 59–66. doi: 10.1104/pp.94.1.59 16667719 PMC1077189

[5] Rothfuss F., Conrad R., 1992. Vertical profiles of CH_4_ concentrations, dissolved substrates and processes involved in CH_4_ production in a flooded Italian rice field. Biogeochemistry 18, 137–152. 10.1007/BF00003274.

[6] Green S.M., 2013. Ebullition of methane from rice paddies: the importance of furthering understanding. Plant Soil 370, 31–34. 10.1007/s11104-013-1790-1.

[7] Cicerone R.J., Shetter J.D., 1981. Sources of atmospheric methane: Measurements in rice paddies and a discussion. J. Geophys. Res. 86, 7203–7209. 10.1029/JC086iC08p07203.

[8] Das K., Baruah K.K., 2008. Methane emission associated with anatomical and morphophysiological characteristics of rice (*Oryza sativa*) plant. Physiologia Plantarum 134, 303–312. doi: 10.1111/j.1399-3054.2008.01137.x 18507814

[9] Holzapfel-Pschorn A., Conrad R., Seiler W., 1986. Effects of vegetation on the emission of methane from submerged paddy soil. Plant Soil 92, 223–233. 10.1007/BF02372636.

[10] Holzapfel-Pschorn A., Seiler W., 1986. Methane emission during a cultivation period from an Italian rice paddy. J. Geophys. Res. 91, 11803–11814. 10.1029/JD091iD11p11803.

[11] IPCC, 1996. Revised 1996 IPCC Guidelines for National Greenhouse Gas Inventories, Reference Manual, Vol. 3, Agriculture, 4.3. https://www.ipcc-nggip.iges.or.jp/public/gl/invs6c.html.

[12] Cheng W., Yagi K., Sakai H., Kobayashi K., 2006. Effects of Elevated Atmospheric CO_2_ Concentrations on CH_4_ and N_2_O Emission from Rice Soil: An Experiment in Controlled-environment Chambers. Biogeochemistry 77, 351–373. 10.1007/s10533-005-1534-2.

[13] Zou J., Huang Y., Jiang J., Zheng X., Sass R.L., 2005. A 3-year field measurement of methane and nitrous oxide emissions from rice paddies in China: Effects of water regime, crop residue, and fertilizer application. Global Biogeochem Cycles 19. 10.1029/2004GB002401.

[14] Meijide A., Manca G., Goded I., Magliulo V., di Tommasi P., Seufert G., et al., 2011. Seasonal trends and environmental controls of methane emissions in a rice paddy field in Northern Italy. Biogeosciences 8, 3809–3821. 10.5194/bg-8-3809-2011.

[15] Simmonds M.B., Anders M., Adviento-Borbe M.A., van Kessel C., McClung A., Linquist B.A., 2015. Seasonal Methane and Nitrous Oxide Emissions of Several Rice Cultivars in Direct-Seeded Systems. J Environ Qual 44, 103–114. doi: 10.2134/jeq2014.07.0286 25602325

[16] IGAC, 1994. Global Measurement Standardization of Methane Emissions from Irrigated Rice Cultivation: A Report of the Rice Cultivation and Trace Gas Exchange Activity (RICE) of the International Global Atmospheric Chemistry (IGAC) Project. IGAC Core Project Office, Massachusetts Institute of Technology, Cambridge, USA.

[17] Pathak H., Aggarwal P.K., Singh S.D., 2012. Climate Change Impact, Adaptation and Mitigation in Agriculture: Methodology for Assessment and Application. Indian Agricultural Research Institute, New Delhi, pp. 1–302. https://cgspace.cgiar.org/handle/10568/42048.

[18] Minamikawa K., Tokida T., Sudo S., Padre A., Yagi K., 2015. Guidelines for measuring CH_4_ and N_2_O emissions from rice paddies by a manually operated closed chamber method. National Institute for Agro-Environmental Sciences, Tsukuba, Japan. http://www.naro.affrc.go.jp/archive/niaes/techdoc/mirsa_guidelines.pdf.

[19] Butterbach-Bahl K., Papen H., Rennenberg H., 1997. Impact of gas transport through rice cultivars on methane emission from rice paddy fields. Plant Cell Environ 20, 1175–1183. 10.1046/j.1365-3040.1997.d01-142.x.

[20] Parkin T.B. and Venterea R.T., 2010. Sampling Protocols. Chapter 3. Chamber-Based Trace Gas Flux Measurements. In: Follett R.F. (Ed.), p. 3–1 to 3–39. https://www.ars.usda.gov/research/publications/publication/?seqNo115=270017.

[21] Rolston D.E., 1986. Gas flux. In: Klute A(Ed.), Methods of Soil Analysis, part 1, 2nd ed., Agron. Monogr. 9. ASA and SSSA, Madison, WI, pp. 1103–1119. 10.2136/sssabookser5.1.2ed.c47.

[22] Moore T.R., Roulet N.T., 1991. A comparison of dynamic and static chambers for methane emission measurements from subarctic fens. Atmos Ocean 29, 102–109. 10.1080/07055900.1991.9649395.

[23] Buendia L.V., Neue H.U., Wassmann R., Lantin R.S., Javellana A.M., Arah J., et al. 1998. An efficient sampling strategy for estimating methane emission from rice field. Chemosphere 36, 395–407. 10.1016/S0045-6535(97)00283-X.

[24] Minamikawa K., Yagi K., Tokida T., Sander B.O., Wassmann R., 2012. Appropriate frequency and time of day to measure methane emissions from an irrigated rice paddy in Japan using the manual closed chamber method. Greenh Gas Meas Manag 2, 118–128. 10.1080/20430779.2012.729988.

[25] Pihlatie M.K., Christiansen J.R., Aaltonen H., Korhonen J.F.J., Nordbo A., Rasilo T., et al. 2013. Comparison of static chambers to measure CH_4_ emissions from soils. Agr Forest Meteorol 171, 124–136. 10.1016/j.agrformet.2012.11.008.

[26] Denmead O.T., Raupach M.R., 1993. Methods for Measuring Atmospheric Gas Transport in Agricultural and Forest Systems. In: Harper L.A., Mosier A.R., Duxbury J.M., Rolston D.E. (Eds.), ASA Special Publications. American Society of Agronomy, Crop Science Society of America, and Soil Science Society of America, Madison, WI, USA, 19–43. 10.2134/asaspecpub55.c2.

[27] Matthias A.D., Peralta Hernández A.R., 1998. Modeling temperatures in soil under an opaque cylindrical enclosure. Agr Forest Meteorol 90, 27–38. 10.1016/S0168-1923(97)00094-4.

[28] Christiansen J.R., Korhonen J.F.J., Juszczak R., Giebels M., Pihlatie M., 2011. Assessing the effects of chamber placement, manual sampling and headspace mixing on CH4 fluxes in a laboratory experiment. Plant Soil 343, 171–185. 10.1007/s11104-010-0701-y.

[29] Juszczak R., 2013. Biases in methane chamber measurements in peatlands. Int Agrophys 27, 159–168. 10.2478/v10247-012-0081-z.

[30] KMA, 2018. Korea meteorological administration. Monthly Report of Automatic Weather System Data. Korea Meteorological Administration, Seoul, Korea (in Korean). https://data.kma.go.kr/data/grnd/selectAsosRltmList.do?pgmNo=36.

[31] RDA, 2010. Fertilization Standards to Crop Plants.National Institute of Agricultural Science and Technology, RDA, Suwon, p. 16 (in Korean).

[32] Blake G. R., Hartge K. H., 1986. Particle density. Methods of soil analysis: Part 1 physical and mineralogical methods, 5, 377–382. 10.2136/sssabookser5.1.2ed.c14.

[33] RDA, 1995. Standard Investigation Methods for Agriculture Experiment. National Institute of Agricultural Science and Technology, RDA, Suwon (in Korean).

[34] Singh S., Singh J.S., Kashyap A.K., 1999. Methane flux from irrigated rice fields in relation to crop growth and N-fertilization. Soil Biol Biochem. 31, 1219–1228. 10.1016/S0038-0717(99)00027-9.

[35] Jones W.J., Nagle D.P., Whitman W.B., 1987. Methanogens and the diversity of archaebacteria. Microbiological Reviews 51, 135–177. doi: 10.1128/mr.51.1.135-177.1987 3104748 PMC373095

[36] Le Mer J., Roger P., 2001. Production, oxidation, emission and consumption of methane by soils: A review. Eur J Soil Biol 37, 25–50. 10.1016/S1164-5563(01)01067-6.

[37] Neue H.-U., 1993. Methane Emission from Rice Fields. BioScience 43, 466–474. 10.2307/1311906.

[38] Wang Z.P., DeLaune R.D., Patrick W.H., Masscheleyn P.H., 1993. Soil Redox and pH Effects on Methane Production in a Flooded Rice Soil. Soil Sci Soc Am J 57, 382–385. 10.2136/sssaj1993.03615995005700020016x.

[39] Kanno T., Miura Y., Tsuruta H., Minami K., 1997. Methane emission from rice paddy fields in all of Japanese prefecture. Nutr Cycl Agroecosys 49, 147–151. 10.1023/A:100977851 7545.

[40] Topp E., Pattey E., 1997. Soils as sources and sinks for atmospheric methane. Can J Soil Sci 77, 167–177. 10.4141/S96-107.

[41] Lu W.F., Chen W., Duan B.W., Guo W.M., Lu Y., Lantin R.S., et al. 2000. Methane emissions and mitigation options in irrigated rice fields in southeast China. Nutr. Cycl. Agroecosys. 58, 65–73. 10.1007/978-94-010-0898-3_6.

[42] Liu C.W., Wu C.Y., 2004. Evaluation of methane emissions from Taiwanese paddies. Sci Total Environ 333, 195–207. doi: 10.1016/j.scitotenv.2004.05.013 15364529

[43] Chun J.A., Shim K.M., Min S.H., Wang Q., 2016. Methane mitigation for flooded rice paddy systems in South Korea using a process-based model. Paddy Water Environ 14, 123–129. 10.1007/s10333-015-0484-0.

[44] Schütz H., Seiler W., Conrad R., 1990. Influence of soil temperature on methane emission from rice paddy fields. Biogeochemistry 11, 77–95. 10.1007/BF00002060.

[45] Sass R.L., Fisher F.M., Turner F.T., Jund M.F., 1991. Methane emission from rice fields as influenced by solar radiation, temperature, and straw incorporation. Global Biogeochem Cycles 5, 335–350. 10.1029/91GB02586.

[46] Yagi K., Minami K., 1993. Spatial and Temporal Variations of Methane Flux from a Rice Paddy Field. In: Oremland R.S. (Ed.), Biogeochemistry of Global Change. Chapmen & Hall, New York, USA, pp. 353–368. 10.1007/978-1-4615-2812-8_19.

[47] Mikkelä C., Sundh I., Svensson B. H., Nilsson M., 1995. Diurnal variation in methane emission in relation to the water table, soil temperature, climate and vegetation cover in a Swedish acid mire. Biogeochemistry 28, 93–114. 10.1007/BF02180679.

[48] Huang Y., Sass R., Fisher F., 1997. Methane emission from Texas rice paddy soils. 2. Seasonal contribution of rice biomass production to CH_4_ emission. Global Change Biology 3, 491–500. 10.10‌46/j.1365-2486.1997.00106.x.

[49] Aulakh M.S., Wassmann R., Bueno C., Kreuzwieser J., Rennenberg H., 2001. Characterization of Root Exudates at Different Growth Stages of Ten Rice (Oryza sativa L.) Cultivars. Plant Biol 3, 139–148. 10.1055/s-2001-12905.

[50] Bacilio-Jiménez M., Aguilar-Flores S., Ventura-Zapata E., Pérez-Campos E., Bouquelet S., Zenteno E., 2003. Chemical characterization of root exudates from rice (Oryza sativa) and their effects on the chemotactic response of endophytic bacteria. Plant Soil, 249(2), 271–277. https://link.springer.com/article/10.1023/A:1022888900465.

[51] Aulakh M.S., Wassmann R., Bueno C., Rennenberg H., 2001. Impact of root exudates of different cultivars and plant development stages of rice (Oryza sativa L.) on methane production in a paddy soil. Plant Soil 230, 77–86. https://dio.org/10.1023/A:1004817212321.

[52] Yu K.W., Wang Z.P., Chen G.X., 1997. Nitrous oxide and methane transport through rice plants. Biology and Fertility of Soils 24, 341–343. 10.1007/s003740050254.

[53] Colmer T.D., 2003. Long-distance transport of gases in plants: a perspective on internal aeration and radial oxygen loss from roots. Plant Cell Environ 26, 17–36. 10.1046/j.1365-3040.2003.00846.x.

[54] Armstrong J., Armstrong W., 1988. Phragmites australis—A preliminary study of soil-oxidizing sites and internal gas transport pathways. New Phytol 108, 373–382. 10.1111/j.14698137.1988.tb04177.x.

