# Supplementary material for: Uncertainty of methane emissions coming from the physical volume of plant biomass inside the closed chamber was negligible during cropping period
Source: PLoS One. 2021 Sep 20;16(9):e0256796. doi: 10.1371/journal.pone.0256796 (PMC8452067; doi:10.1371/journal.pone.0256796)
Supplement: S1 Fig — (DOCX) [file pone.0256796.s001.docx]

**Supplementary Figure-1**. Changes in air temperatures inside and outside the closed chamber during rice cropping period.
