# Supplementary material for: Uncertainty of methane emissions coming from the physical volume of plant biomass inside the closed chamber was negligible during cropping period
Source: PLoS One. 2021 Sep 20;16(9):e0256796. doi: 10.1371/journal.pone.0256796 (PMC8452067; doi:10.1371/journal.pone.0256796)
Supplement: S2 Fig — (DOCX) [file pone.0256796.s002.docx]

**Supplementary Figure-2**. Changes in plant growth characteristics during rice cropping season.
