# Supplementary material for: Uncertainty of methane emissions coming from the physical volume of plant biomass inside the closed chamber was negligible during cropping period
Source: PLoS One. 2021 Sep 20;16(9):e0256796. doi: 10.1371/journal.pone.0256796 (PMC8452067; doi:10.1371/journal.pone.0256796)
Supplement: S3 Fig — (DOCX) [file pone.0256796.s003.docx]

| 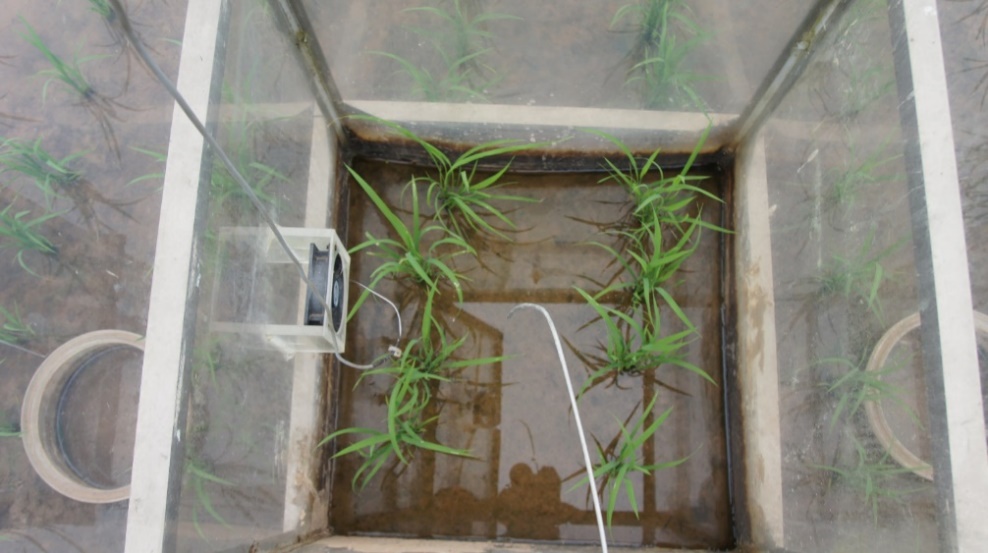 | 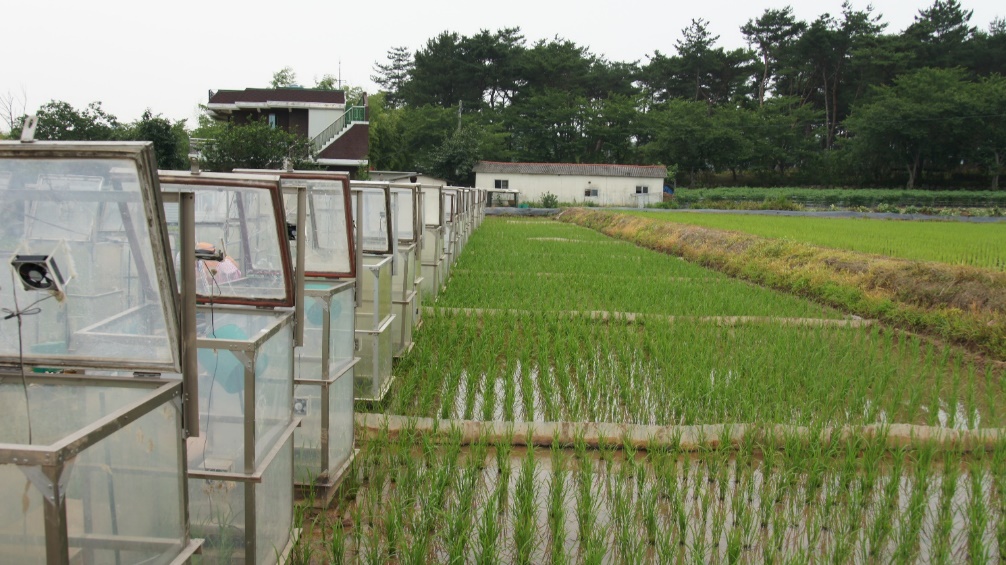 |
| --- | --- |
| 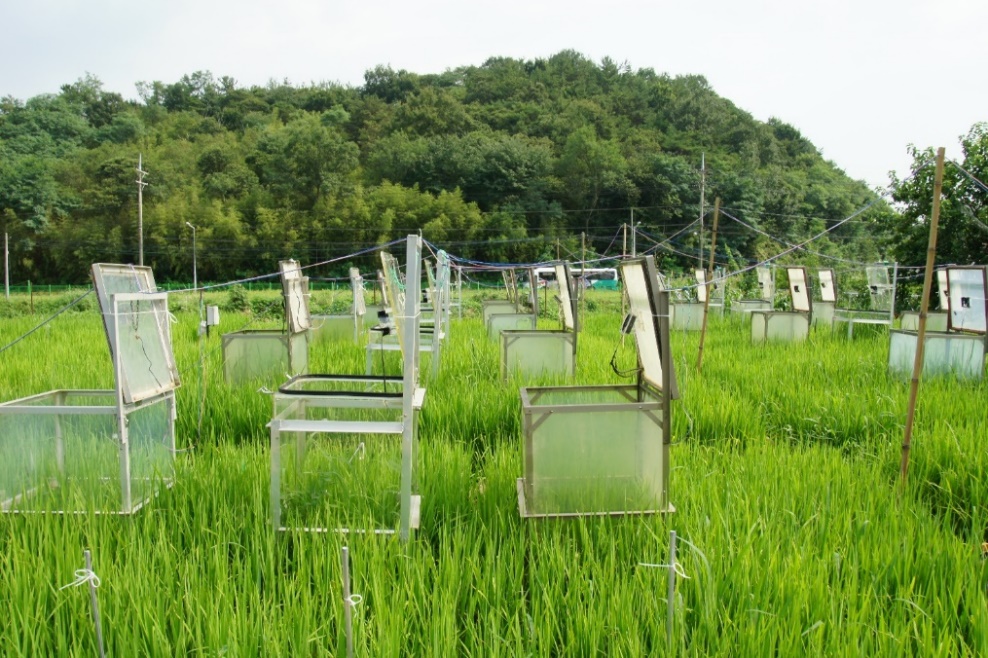 | 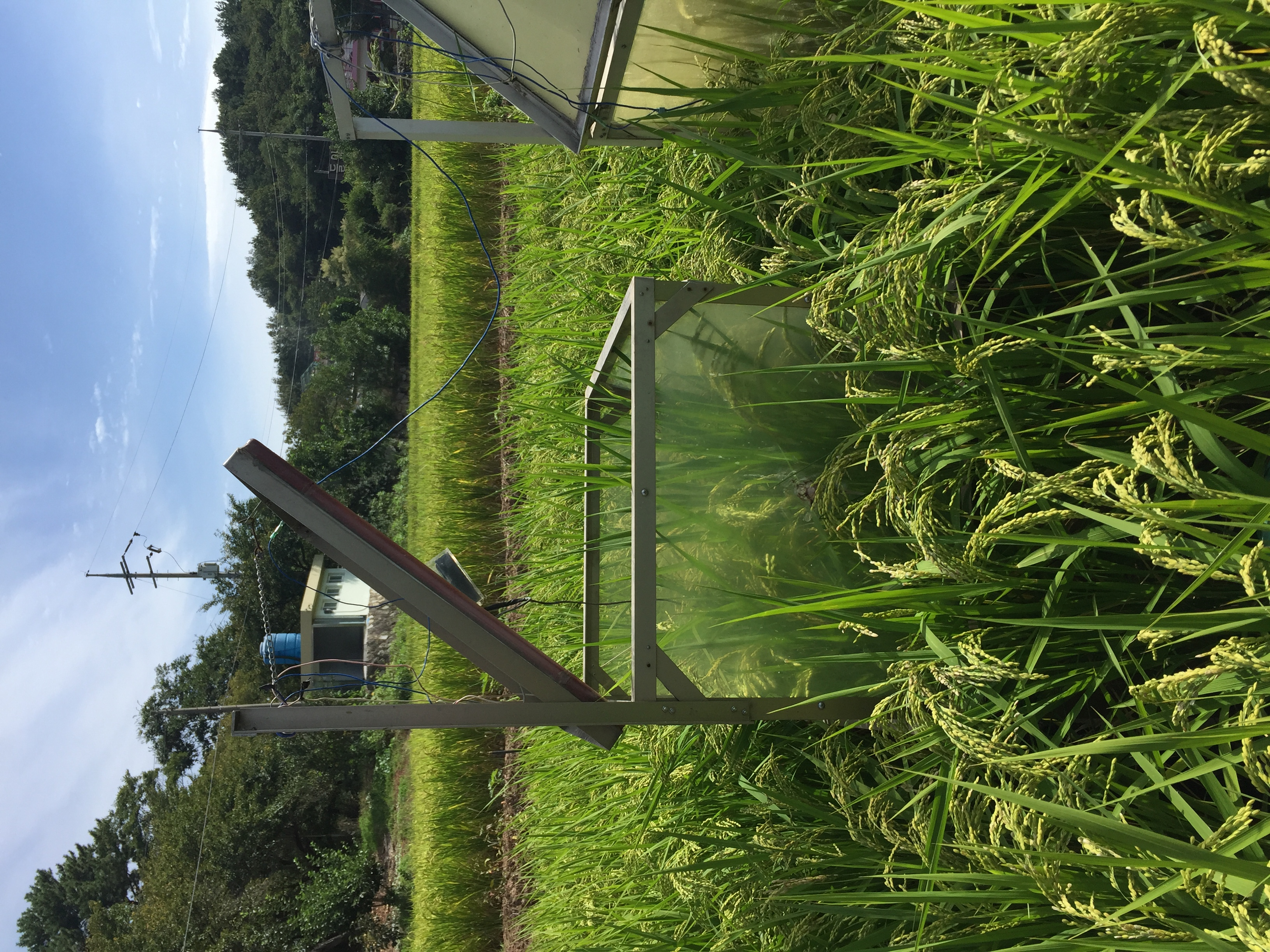 |

**Supplementary Figure-3**. Static chamber installation and rice plant growth inside the closed chamber
