# Supplementary material for: Uncertainty of methane emissions coming from the physical volume of plant biomass inside the closed chamber was negligible during cropping period
Source: PLoS One. 2021 Sep 20;16(9):e0256796. doi: 10.1371/journal.pone.0256796 (PMC8452067; doi:10.1371/journal.pone.0256796)
Supplement: S1 Table — (DOCX) [file pone.0256796.s004.docx]

**Supplementary Table 1**. The maximum plant volumes and growth characteristics with statistical significance between rice cultivars

|  | Plant volume | Tiller no. | Plant height | Fresh weight | Dry weight |
| --- | --- | --- | --- | --- | --- |
|  | cm^3^ | No. hill^-1^ | cm | g hill^-1^ | g hill^-1^ |
| Chuchung | 325.6$\pm$30.2^a^ | 19.7$\pm$2.9^a^ | 106.3$\pm$3.8^a^ | 192.9$\pm$18.9^a^ | 49.3$\pm3$^ab^ |
| Dongjin | 309.3$\pm$19.2^a^ | 15.7$\pm$1.2^bc^ | 95.9$\pm$4.7^a^ | 162.9$\pm$8.8^a^ | 39.4$\pm$1.9^a^ |
| Ilmi | 351.9$\pm$43.9^a^ | 15$\pm$1.6^abc^ | 100.8$\pm$2.3^ab^ | 206.1$\pm$16.6^a^ | 62.9$\pm$5.6^bc^ |
| Junam | 329.2$\pm$38.1^a^ | 18.7$\pm$1.2^ab^ | 95.6$\pm$3.0^b^ | 210$\pm$22^a^ | 69.2$\pm$6.4^c^ |
| Saenuri | 347.7$\pm$11.7^a^ | 11.7$\pm$1.2^c^ | 125$\pm$4.1^c^ | 189.9$\pm$7.5^a^ | 47$\pm$4.1^a^ |

Different letters represent a significant difference (P<0.05).
